# Alterations of metabolic activity in human osteoarthritic osteoblasts by lipid peroxidation end product 4-hydroxynonenal

**DOI:** 10.1186/ar2066

**Published:** 2006-10-16

**Authors:** Qin Shi, France Vaillancourt, Véronique Côté, Hassan Fahmi, Patrick Lavigne, Hassan Afif, John A Di Battista, Julio C Fernandes, Mohamed Benderdour

**Affiliations:** 1 Orthopaedic Research Laboratory, Sacre-Coeur Hospital, University of Montreal, 5400 Gouin West, Montreal, Quebec, Canada H4J 1C5

## Abstract

4-Hydroxynonenal (HNE), a lipid peroxidation end product, is produced abundantly in osteoarthritic (OA) articular tissues, but its role in bone metabolism is ill-defined. In this study, we tested the hypothesis that alterations in OA osteoblast metabolism are attributed, in part, to increased levels of HNE. Our data showed that HNE/protein adduct levels were higher in OA osteoblasts compared to normal and when OA osteoblasts were treated with H_2_O_2_. Investigating osteoblast markers, we found that HNE increased osteocalcin and type I collagen synthesis but inhibited alkaline phosphatase activity. We next examined the effects of HNE on the signaling pathways controlling cyclooxygenase-2 (COX-2) and interleukin-6 (IL-6) expression in view of their putative role in OA pathophysiology. HNE dose-dependently decreased basal and tumour necrosis factor-α (TNF-α)-induced IL-6 expression while inducing COX-2 expression and prostaglandin E_2 _(PGE_2_) release. In a similar pattern, HNE induces changes in osteoblast markers as well as PGE_2 _and IL-6 release in normal osteoblasts. Upon examination of signaling pathways involved in PGE_2 _and IL-6 production, we found that HNE-induced PGE_2 _release was abrogated by SB202190, a p38 mitogen-activated protein kinase (MAPK) inhibitor. Overexpression of p38 MAPK enhanced HNE-induced PGE_2 _release. In this connection, HNE markedly increased the phosphorylation of p38 MAPK, JNK2, and transcription factors (CREB-1, ATF-2) with a concomitant increase in the DNA-binding activity of CRE/ATF. Transfection experiments with a human COX-2 promoter construct revealed that the CRE element (-58/-53 bp) was essential for HNE-induced COX-2 promoter activity. However, HNE inhibited the phosphorylation of IκBα and subsequently the DNA-binding activity of nuclear factor-κB. Overexpression of IKKα increased TNF-α-induced IL-6 production. This induction was inhibited when TNF-α was combined with HNE. These findings suggest that HNE may exert multiple effects on human OA osteoblasts by selective activation of signal transduction pathways and alteration of osteoblastic phenotype expression and pro-inflammatory mediator production.

## Introduction

Lipid peroxidation (LPO) is a process initiated by lipid reaction with reactive oxygen species (ROS). ROS are generated during normal cellular metabolism or under oxidative stress stimuli (for example, cytokine and UV radiation). Polyunsaturated fatty acids of cellular membrane lipids are targets of ROS attack and undergo LPO, leading to the formation of chemically reactive lipid aldehydes capable of diffusing from their site of origin. Similar to ROS, aldehydes can cause severe damage to nucleic acids and proteins, altering their functions and leading to the loss of both structural and metabolic function of cells. Under intense oxidative stress, aldehyde levels increase and take part in numerous pathological conditions such as cancer, arthritis, arthrosclerosis, and cardiac diseases[1]. 4-Hydroxynonenal (HNE) is the principal α, β-unsaturated aldehyde formed from LPO of both ω-3 and ω-6 polyunsaturated fatty acids[2]. The accumulation of HNE exhibits a wide range of biological activities, including stimulation of neutrophil migration, mitochondrial enzyme inhibition, and activation of stress-signaling pathways via transcription factors and protein kinase pathways, as well as inhibition of the nuclear factor-κB (NF-κB) signaling pathway [3-6].

Osteoarthritis (OA) is a degenerative disease characterised by a progressive degradation of articular cartilage accompanied with secondary inflammation of synovial membranes. Although major progress has been made in the last few years, the aetiology, pathogenesis, and progression of this disease are not fully understood. Recent clinical and research findings suggest that oxidative stress-induced LPO products can play an important role in the pathogenesis of OA. Grigolo and colleagues [7] were the first to demonstrate that the formation of HNE and malondialdehyde (MDA) is enhanced in synoviocytes from patientswith OA. In a recent study, we have shown that HNE level is higher in synovial fluids of patients with OA compared with normal subjects and in human articular OA chondrocytes exposed to ROS donors. In addition, we have reported novel mechanisms linking HNE to OA cartilage degradation. These mechanisms emphasise the implication of HNE in transcriptional and post-translational modifications of type II collagen and matrix metalloproteinase-13 in human OA chondrocytes, and result in cartilage extracellular matrix (ECM) degradation[8]. However, little is known about the role of HNE in bone.

Abnormal subchondral trabecular bone remodelling is present in patients with OA. The increased stiffness of OA bone with subchondral bone plate sclerosis results in increased trabecular thickening and decreased trabecular space volume/bone mineralisation with the bone cell defects[9]. Type 1 collagen (Col I) and other specific osteoblast phenotypic markers, such as osteocalcin (OC) and alkaline phosphatase (ALPase), are released from osteoblasts during bone formation[10]. It is believed that alterations in osteoblast metabolism play an important role in this disease by producing excess bone-resorbing cytokines and prostaglandins[11]. Among the pro-inflammatory mediators, interleukin-6 (IL-6) is a multifunctional cytokine involved in osteoclast recruitment and differentiation into mature osteoclast. Prostaglandin E_2 _(PGE_2_), produced primarily by cyclooxygenase-2 (COX-2) [12], plays an important role in the local regulation of bone formation and bone resorption[13]. These biologically active mediators are also considered to be biochemical markers of bone metabolism and are regulated in bone by pro-inflammatory cytokines.

The objective of this study was to investigate the role of HNE in OA osteoblast metabolism by determining its effect on the production of biological markers and pro-inflammatory mediators. Furthermore, we explored the signaling pathways involved in HNE-regulated IL-6 and PGE_2 _production.

## Materials and methods

### Osteoblast culture

Normal human osteoblasts were purchased from PromoCell GmbH (Heidelberg, Germany) and were cultured according to the manufacturer's specifications. OA osteoblasts were isolated from trabecular bone specimens from patients suffering from advanced OA and undergoing primary total knee replacement. The experimental protocol was approved by the Research Ethics Board at Sacre-Cæur Hospital of Montreal. The osteoblast cell cultures were prepared as already described[11]. Briefly, trabecular bone samples were cut into small pieces of 2 mm^2 ^prior to their sequential digestion in the presence of 1 mg/ml collagenase type I (Sigma-Aldrich Canada Ltd., Oakville, ON, Canada) in BGJb media (Invitrogen Canada Inc., Burlington, ON, Canada) without serum at 37°C for 30, 30, and 240 minutes. After being washed with the same media, the digested bone pieces were cultured in 25 cm^2 ^plastic cell culture flasks (Corning Incorporated, Corning, NY, USA) with BGJb media containing 20% foetal bovine serum (FBS) (Invitrogen Life Technologies). This medium was replaced every 2 days until cell outgrowths appeared around the explants. At confluence, cells were split once and plated at 50,000 cells per cm^2 ^in culture plates (Falcon, Lincoln Park, NJ, USA) with Ham's F-12/Dulbecco's modified Eagle's medium (HAMF-12/DMEM) (Sigma-Aldrich Canada Ltd.) containing 10% FBS and 50 mg/ml ascorbic acid and grown to confluence again. Only first-passage cells were used in our experiments.

### HNE assay

Normal and OA osteoblasts were incubated for 24 hours with or without increasing concentrations of H_2_O_2 _(1 to 100 μM). Total cellular levels of HNE/protein adducts were assessed in cellular extracts of osteoblasts using an in-house enzyme-linked immunosorbent assay (ELISA) as previously described [5].

### Determination of OC level and ALPase activity

Osteoblasts were incubated for 24 hours in HAMF-12/DMEM containing 2% charcoal-stripped FBS, which yields maximal stimulation of ALPase activity and OC secretion. Cells were then incubated for 48 hours in the same medium in the presence of increasing concentrations of HNE (0 to 20 μM). The medium was collected at the end of the incubation and frozen at -80°C prior to assay. Cells were washed twice with phosphate-buffered saline, pH 7.4, and solubilised in ALPase buffer (100 mM glycine, 1 mM MgCl_2_, 1 mM ZnCl_2_, 1% Triton X-100; pH 10.5) for 60 minutes with agitation at 4°C. Cellular ALPase activity was determined as the release of p-nitrophenol hydrolysed from p-nitrophenyl phosphate (12.5 mM final concentration) at 37°C for 30 minutes after cell solubilisation in ALPase buffer as described above. Protein determination was performed by the bicinchoninic acid method[14]. Nascent OC was determined by a specific enzyme immunoassay (Biomedical Technologies, Inc., Stoughton, MA, USA). The detection limit of this assay is 0.5 ng/ml, and 2% charcoal-treated FBS contains less than 0.1 ng/ml of OC.

### IL-6 and PGE_2 _assays

For the HNE dose-response curves, osteoblasts were incubated in 0.5% FBS/HAMF-12/DMEM for 48 hours with increasing concentrations of HNE (0 to 20 μM). After incubation, the culture medium was collected and the IL-6 and PGE_2 _levels were determined using specific commercial kits from R&D Systems, Inc. (Minneapolis, MN, USA) and Cayman Chemical Company (Ann Arbor, MI, USA), respectively, according to the manufacturers' specifications. The sensitivities of the assays were 3 and 9 pg/ml, respectively.

### Protein detection by Western blotting

Osteoblasts were incubated in fresh medium containing 0.5% FBS/HAMF12/DMEM in the presence of increasing concentrations of HNE (0 to 20 μM) for 24 hours or in the presence of 20 μM of HNE for increasing periods of incubation. Twenty to 50 μg of cellular protein extract was subjected to discontinuous 4% to 12% SDS-PAGE under reducing conditions and transferred onto nitrocellulose membrane (Bio-Rad Laboratories, Inc., Hercules, CA, USA). The membranes were immersed overnight at 4°C in a blocking solution consisting of TTBS (20 mM Tris, pH7.4, 150 mM NaCl, 0.1% Tween 20) and 5% skim milk and incubated again overnight in blocking buffer containing the polyclonal rabbit anti-COX-2 or anti-Col I (1:1,000 dilution; Oncogene Research Products, San Diego, CA, USA). The membranes were then washed three times with TTBS and incubated for 1 hour at 22°C with the second antibody (anti-rabbit immunoglobulin G-horse radish peroxidase; New England Biolabs Ltd., Mississauga, ON, Canada) and washed again. Detection was carried out using Supersignal west dura extended duration substrate (Pierce Biotechnology, Inc., Rockford, IL, USA). Membranes were prepared for autoradiography and exposed to clear-blue x-ray film (Pierce) and then subjected to a digital imaging system (Bio-Rad Laboratories, Inc.). For the total and phosphorylated level of mitogen-activated protein kinases (MAPKs) (p38, c-Jun NH_2_-terminal kinase [JNK] 1/2, and extracellular signal-regulated kinase [ERK] 1/2) as well as transcription factors (activating transcription factor-2 [ATF-2], CRE-binding factor-1 [CREB-1], and IκBα), we used specific PhosphoPlus kits (New England Biolabs Ltd.).

### Real-time quantitative reverse transcriptase-polymerase chain reaction

Total RNA was extracted from OA osteoblasts using TRIzol^® ^reagent (Invitrogen Life Technologies) according to the manufacturer's recommendations. The RNA was quantitated using the RiboGreen RNA quantitation kit (Molecular Probes, now part of Invitrogen, Carlsbad, CA, USA), dissolved in RNase-free H_2_O, and stored at -80°C until use. One microgram of total RNA was reverse-transcribed using Moloney murine leukaemia virus reverse transcriptase (Fermentas Canada Inc., Burlington, ON, Canada) as detailed in the manufacturer's guidelines. One fiftieth of the reverse transcriptase reaction was analysed by real-time quantitative polymerase chain reaction (PCR). The nucleotide sequence of primers are shown below:

ALPase [15]: 5'-CCCAAAGGCTTCTTCTTG-3' (sense)

5'-CTGGTAGTTGTTGTGAGCAT-3' (anti-sense),

OC [15]: 5'-ATGAGAGCCCTCACACTCCTC-3' (sense)

5'-GCCGTAGAAGCGCCGATAGGC-3' (anti-sense),

Col I α 1 [16]: 5' CATCCTCGACGGCATCTCAGC-3' (sense)

5'-TTGGGTCAGGGGTGGTTATTG-3' (anti-sense),

IL-6 [17]: 5'-TTCAAATGAGATTGTGGGAAAATTGCT-3' (sense)

5'-AGTTCATCTCTGCCTGAGTATCTT-3' (anti-sense),

COX-2 [18]: 5'-TTCAAATGAGATTGTGGGAAAATTGCT-3' (sense)

5'-AGTTCATCTCTGCCTGAGTATCTT-3' (anti-sense), and

glyceraldehyde-3-phosphate dehydrogenase (GAPDH) [19]:

5'-CAG AAC ATC ATC CCT GCC TCT-3' (sense)

5'-GCT TGA CAAAGT GGT CGT TGA G-3' (anti-sense).

Quantitative PCR analysis was performed in a total volume of 50 μl containing template DNA, 200 nM of sense and antisense primers, 25 μl of SYBR^® ^Green master mix (Qiagen Inc., Mississauga, ON, Canada), and uracil-N-glycosylase (UNG) (0.5 Units; Epicentre Biotechnologies, Madison, WI, USA). After incubation at 50°C for 2 minutes (UNG reaction) and at 95°C for 10 minutes (UNG inactivation and activation of the AmpliTaq Gold enzyme), the mixtures were subjected to 40 amplification cycles (15 seconds at 95°C for denaturation and 1 minute for annealing and extension at 60°C). Incorporation of SYBR^® ^Green dye into PCR products was monitored in real time using a GeneAmp 5700 Sequence detection system (Applied Biosystems, Foster City, CA, USA) allowing determination of the threshold cycle (C_T_) at which exponential amplification of PCR products begins. After PCR, dissociation curves were generated with one peak, indicating the specificity of the amplification. The C_T _value was obtained from each amplification curve using the software provided by the manufacturer (Applied Biosystems). Preliminary experiments showed that the amplification efficiency of COX-2, ALPase, OC, Col α1, IL-6, and GAPDH was similar.

Relative amounts of mRNA in normal and OA cartilage were determined using the standard curve method. Serial dilutions of internal standards (plasmids containing cDNA of target genes) were included in each PCR run, and standard curves for the target gene and for GAPDH were generated by linear regression using log (C_T_) versus log (cDNA relative dilution). The C_T _values were then converted to number of molecules. Relative mRNA expression in cultured chondrocytes was determined using the ΔΔC_T _method, as detailed in the manufacturer's guidelines (Applied Biosystems). A ΔC_T _value was first calculated by subtracting the C_T _value for the housekeeping gene *GAPDH *from the C_T _value for each sample. A ΔΔC_T _value was then calculated by subtracting the ΔC_T _value of the control (unstimulated cells) from the ΔC_T _value of each treatment. Fold changes compared with the control were then determined by raising 2 to the ΔΔC_T _power. Each PCR reaction generated only the expected specific amplicon as shown by the melting-temperature profiles of the final product and by gel electrophoresis of test PCR reactions. Each PCR was performed in triplicate on two separate occasions for each independent experiment.

### Nuclear extract preparation and electrophoretic mobility shift assay

OA osteoblasts were incubated with HNE alone or in combination with 1 ng/ml tumour necrosis factor-α (TNF-α) for 1 hour. Nuclear extracts were prepared and electrophoretic mobility shift assay (EMSA) was performed as previously described[20]. Double-stranded oligonucleotide probes for CRE (5'-AGAGATTGCCTGACGTCAGAGAGCTAG-3') and NF-κB (5'-AGTTGAGGGGACTT TCCCAGGC-3') were end-labeled with [γ-^32^P]-ATP using a kit (Promega Corporation, Madison, WI, USA). The binding reactions were conducted with 5 μg of nuclear extract and of 2 × 10^5 ^cpm of [γ-^32^P]-labeled oligonucleotide probe at 22°C for 20 minutes in a final volume of 10 μl, and complexes were resolved on non-denaturing 6% polyacrylamide gels. Then, gels were fixed, dried, and exposed to clear-blue x-ray film (Pierce).

Supershift assays were performed as described above with nuclear extracts from cells treated with HNE (20 μM) or TNF-α (1 ng/ml) for 1 hour. Two micrograms of the antibodies were added to the shift reaction mixture 20 minutes after the incubation period, followed by another incubation at 4°C overnight. The antibodies were specific for the transcription factors ATF-2, p65, and p50 (Santa Cruz Biotechnology, Inc., Santa Cruz, CA, USA).

### Plasmids and transient transfections

The human COX-2 promoter constructs used included a wild-type (WT) (-415)-Luciferase (Luc) COX-2 promoter plasmid, mutated ATF/CRE (-58/-53) (-415)-Luc COX-2 promoter plasmid, and mutated NF-κB (-223/-214) (-415)-Luc COX-2 promoter plasmid, as previously described [21]. Expression vectors for WT (pCMV-Flag-p38) and dominant negative (DN) (pCMV-Flag-p38) p38 MAPK were a kind gift from Dr. R.J. Davies (University of Massachusetts). Expression vector for IKKα was generously given by Dr. M. Karin (University of California). A pCMV-β-galactosidase (pCMV-β-gal) reporter vector was purchased from Promega Corporation.

Human MG-63 osteoblast-like line cells (American Type Culture Collection, Manassas, VA, USA) (approximately 50% confluence) were transiently transfected in 12-well cluster plates using lipofectamine 2000™ reagent methods (Invitrogen Life Technologies) according to the manufacturer's protocol. Briefly, transfections were conducted for 6 hours with DNA lipofectamine complexes containing 10 μl of lipofectamine reagent, 2 μg DNA plasmid, and 0.5 μg of pCMV-β-gal (as a control of transfection efficiency). After washing, medium was replaced by a fresh medium containing 1% FBS and experiments were performed in this medium supplemented with the factors under study. For promoter study, Luciferase activity was determined in cellular extracts by a kit (Luciferase Assay System; Promega Corporation) using a microplate luminometer (Applied Biosystems) and normalised to β-gal level, which was quantified by a specific ELISA (Roche Diagnostics Canada, Laval, QC, Canada). To study the effect of p38 MAPK and IKKα overexpression on PGE_2 _and IL-6 production, cells were transfected with the appropriated WT p38 MAPK, DN p38 MAPK, or IKKα expression vector as described above and then culture medium was collected for PGE_2 _and IL-6 assay as described above.

### Statistical analysis

The data are expressed as the mean ± standard error of the mean. Statistical significance was assessed by unpaired Student *t *test, and *P *< 0.05 was considered significant.

## Results

### HNE production in OA osteoblasts

**Figure 1:**
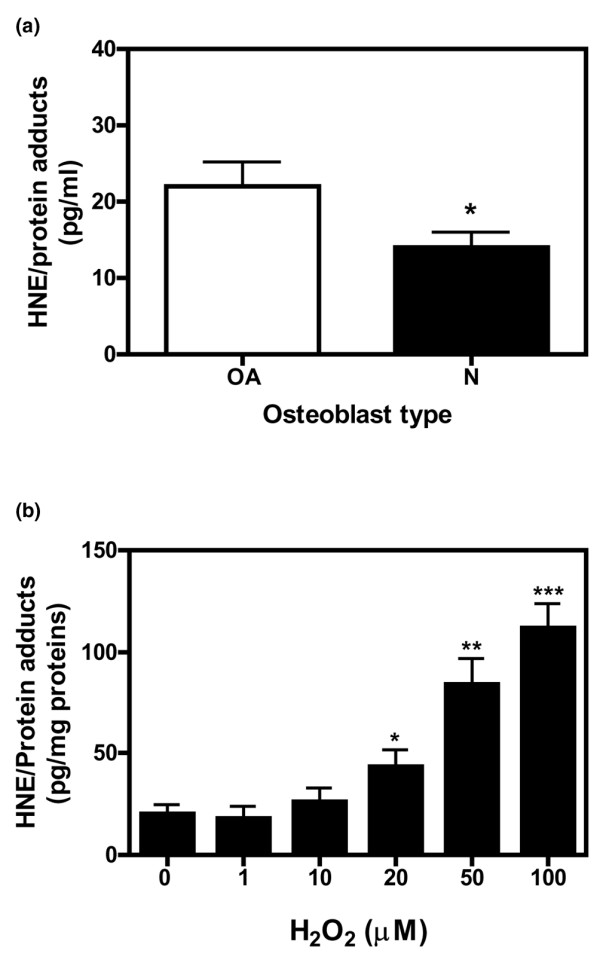
Determination of HNE/protein adduct concentrations in normal (N) and osteoarthritic (OA) osteoblast. HNE/protein adduct levels were measured by enzyme-linked immunosorbent assay in cellular extracts from untreated **(a) **or treated **(b) **osteoblasts with increasing concentrations of H_2_O_2 _for 24 hours at the indicated concentrations. HNE/protein adduct levels were expressed in picograms of HNE/protein adducts per milligrams of total proteins. Data are mean ± standard error of the mean (*n *= 3). Statistics: Student unpaired *t *test; *p < 0.05, **P < 0.01, ***P < 0.001. HNE, 4-hydroxynonenal.

To provide evidence that HNE production was increased during OA development, the level of this aldehyde was determined in cellular extract of normal and OA osteoblasts. As shown in Figure 1a, HNE/protein adduct levels were 1.4-fold higher in OA cells compared with the normal (*p *≤ 0.05). To confirm that OA osteoblasts are able to produce HNE under oxidative stress, cells were incubated for 24 hours with increasing concentrations of H_2_O_2 _and levels of HNE/protein adducts were quantified in cellular extracts. The data showed that H_2_O_2 _at different concentrations induces the formation of HNE/proteins adducts in OA osteoblasts in a dose-dependent manner (Figure 1b).

### Changes in differentiation markers (ALPase and OC) and Col I

**Figure 2:**
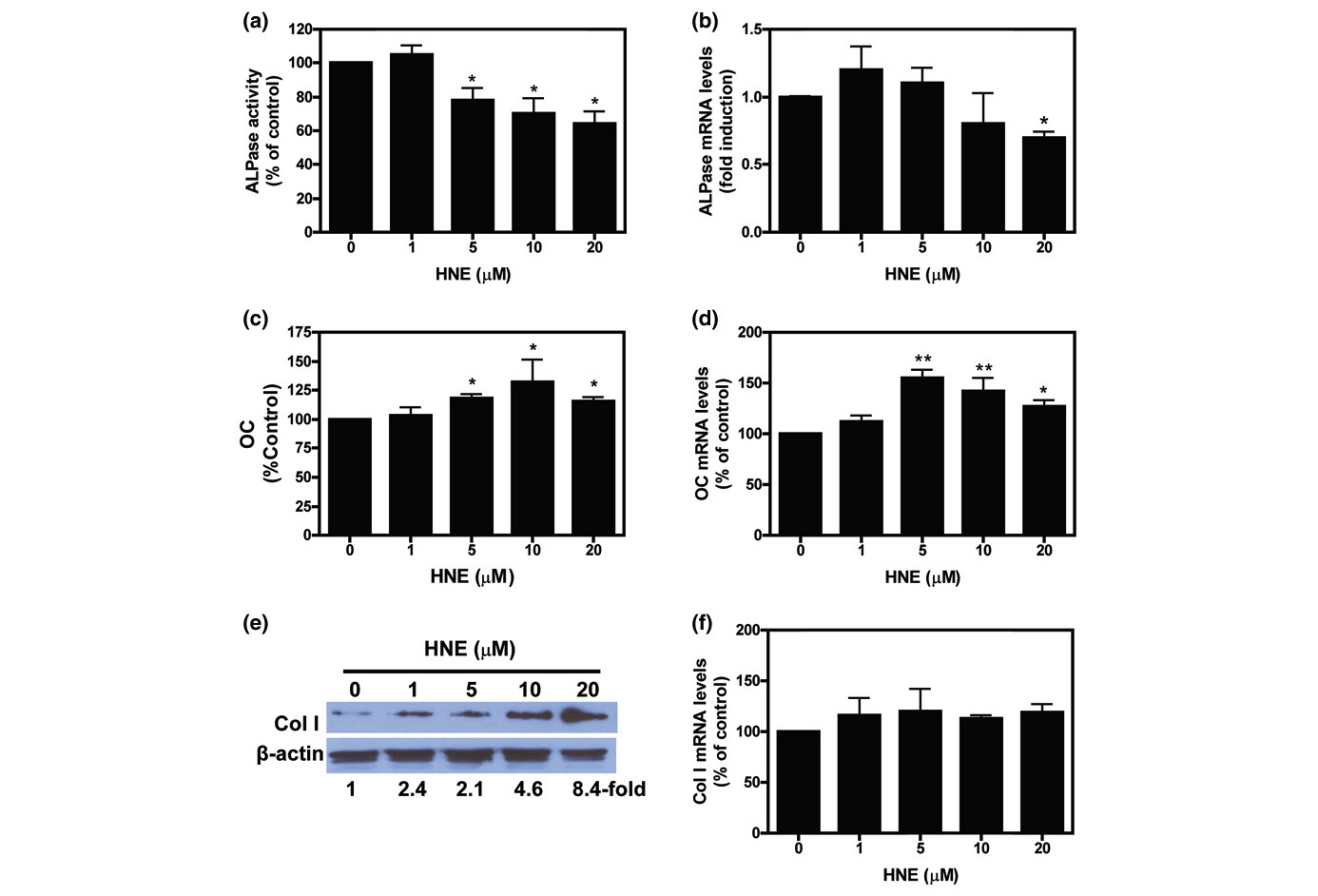
Effect of HNE on osteoblast markers ALPase, OC, and Col I. Human osteoarthritic osteoblasts were incubated with increasing concentrations of HNE for 48 hours and then ALPase activity **(a) **and Col I protein level **(e) **were determined in cellular extract as described in Materials and methods. The OC release **(c) **was determined in culture medium by enzyme-linked immunosorbent assay. For mRNA level, cells were incubated for 4 hours in the absence or presence of indicated concentrations of HNE, total RNA was isolated and reverse-transcribed into cDNA, and ALPase **(b)**, OC **(d)**, and Col I **(f) **were quantified using real-time polymerase chain reaction. All experiments were performed in triplicate, and negative controls without template RNA were included in each experiment as indicated in Materials and methods. mRNA levels were normalised to those of GAPDH (glyceraldehyde-3-phosphate dehydrogenase) mRNA. Data are means ± standard error of the mean of *n *= 3 and expressed as a percentage of untreated cells. Statistics: Student unpaired *t *test; *p < 0.05, **p < 0.01. ALPase, alkaline phosphatase; Col I, type I collagen; HNE, 4-hydroxynonenal; OC, osteocalcin.

Because ALPase, OC, and Col I are the principal biomarkers of osteoblasts and considered to be good indicators for bone formation and metabolic activity, we tested the ability of HNE to alter their expression and, in turn, the phenotype of the osteoblasts. Figure 2 depicts the variation in osteoblast production of ALPase (Figure 2a,b), OC (Figure2c,d), and Col I (Figure 2e,f) after HNE incubation. Compared with control, HNE dose-dependently inhibited significantly osteoblast ALPase activity by 19.6%, 25.4%, and 32.1% (*p *< 0.01) in the presence of 5, 10, and 20 μM of HNE, respectively (Figure 2a). However, ALPase mRNA expression was significantly inhibited only at 20 μM HNE (20%; *p *< 0.05) (Figure 2b).

In contrast to the inhibition of ALPase, OC protein level was increased significantly in the presence of HNE (15%, 25%, and 20% at 5, 10, and 20 μM, respectively; *p *< 0.05) (Figure 2c). OC mRNA levels were also increased at different concentrations of HNE, with a maximum stimulation of 155% at 5 μM HNE (*p *< 0.01) (Figure 2d).

Finally, we explored the effect of HNE on Col I, which constitutes 90% of the total organic ECM in mature bone. Our data showed that HNE increased Col I protein expression by factors of 2.4, 2.1, 4.6, and 8.4 at concentrations of 1, 5, 10, and 20 μM, respectively (Figure 2e), although this inductive effect of HNE was not manifested at the mRNA level (Figure 2f).

### HNE inhibits IL-6 expression

**Figure 3:**
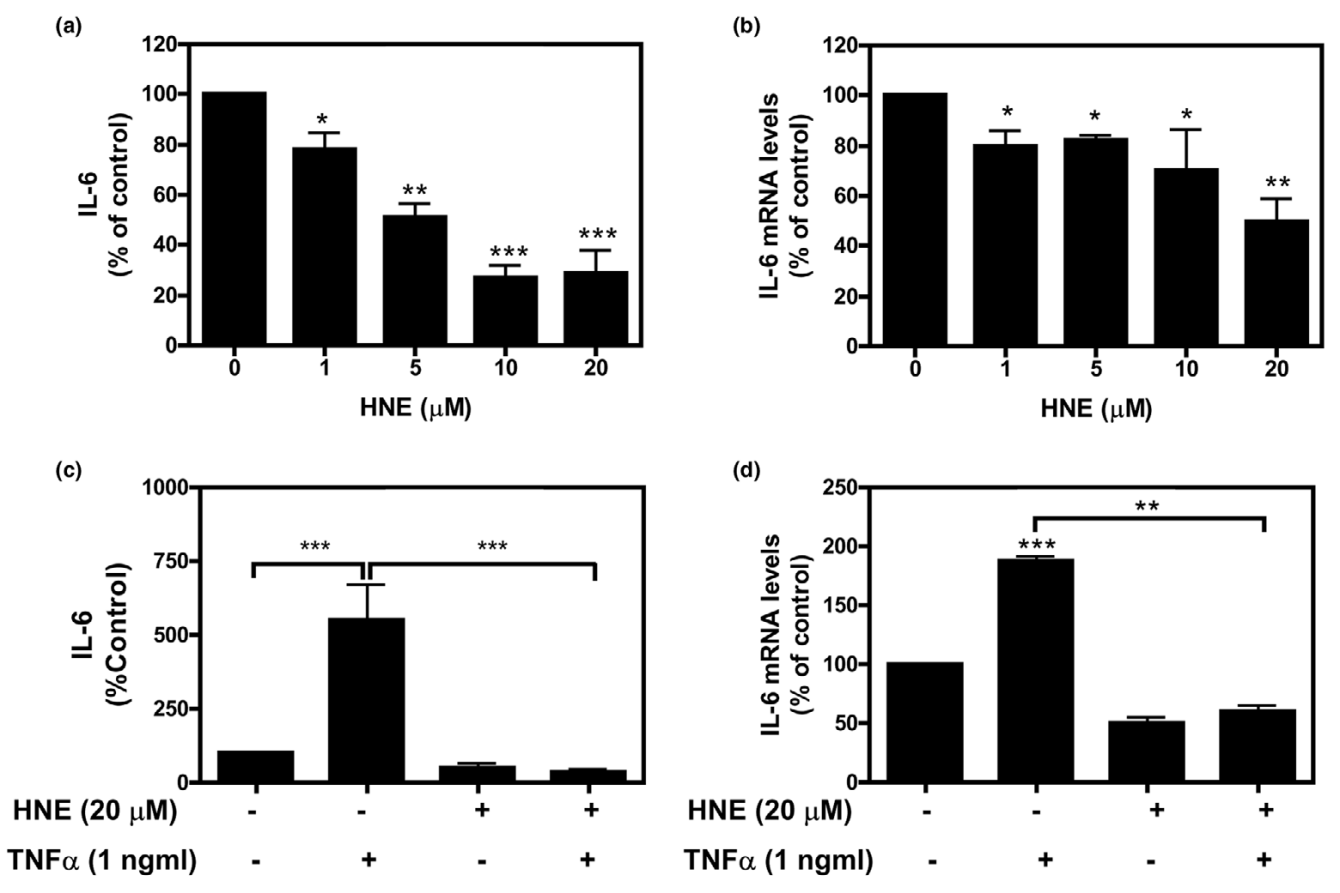
Effect of HNE on IL-6 protein production in osteoblasts. Osteoblasts were treated with HNE (0 to 20 μM) for 48 or 4 hours for IL-6 protein **(a) **(*n *= 7) and mRNA **(b) **(*n *= 3) determination, respectively. The effect of HNE combined with TNF-α was evaluated by incubating osteoblasts with HNE (20 μM) for 30 minutes and subsequently stimulating them with TNF-α (1 ng/ml) for 48 or 4 hours for IL-6 protein **(c) **(*n *= 7) and mRNA **(d) **(*n *= 3) determination, respectively. mRNA levels of each gene were quantified by real-time polymerase chain reaction as described in Materials and methods and normalised to those of GAPDH (glyceraldehyde-3-phosphate dehydrogenase) mRNA. Data are means ± standard error of the mean and expressed as a percentage of untreated cells. Statistics: Student unpaired *t *test; *p < 0.05, **p < 0.01, ***p < 0.001. HNE, 4-hydroxynonenal; IL-6, interleukin-6; TNF-α, tumour necrosis factor-α.

To determine whether HNE is a modulator of IL-6 production, osteoblasts were incubated with 0 to 20 μM of HNE for 48 or 4 hours for IL-6 protein and mRNA determination, respectively. As shown in Figure 3, there was a significant dose-dependent inhibition of IL-6 protein release (Figure 3a) and mRNA expression (Figure 3b) after incubation of osteoblasts with increasing concentrations of HNE. To test the combined effect of HNE with TNF-α, osteoblasts were preincubated with HNE (20 μM) for 30 minutes and then stimulated with TNF-α (1 ng/ml). Compared with untreated cells, TNF-α significantly induced IL-6 release by 549% (Figure 3c). This induction was completely inhibited in the presence of 20 μM HNE (47% of untreated cells). At mRNA level, HNE also showed a significant (approximately 70%) decrease of TNF-α-induced IL-6 mRNA expression (Figure 3d).

### HNE induces PGE_2 _release and COX-2 expression

**Figure 4:**
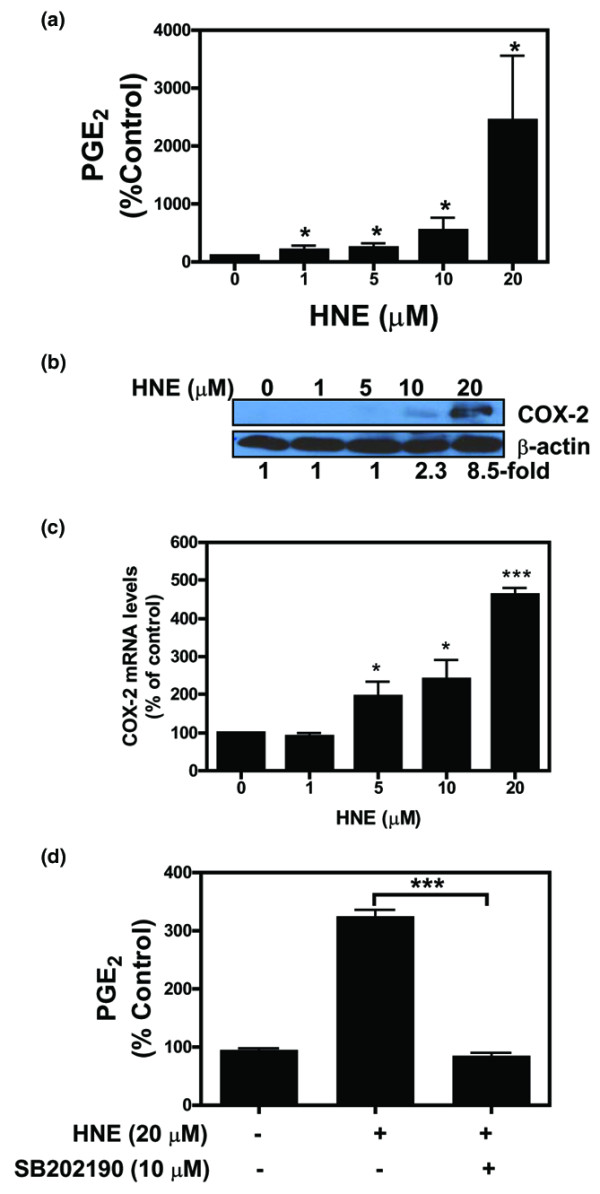
Effect of HNE on PGE_2 _release and COX-2 expression.**(a) **Osteoblasts were treated with HNE (0 to 20 μM) for 48 hours, and PGE_2 _release was evaluated in culture medium by PGE_2 _enzyme immunoassay kit. **(b, c) **Osteoblasts were treated with HNE (0 to 20 μM) for 48 or 4 hours for protein and mRNA determination, respectively. COX-2 protein expression **(b) **and mRNA expression **(c) **were evaluated by Western blot and real-time reverse transcriptase-polymerase chain reaction, respectively. Quantifications of COX-2 protein and mRNA levels were normalised, respectively, to those of β-actin protein and GAPDH (glyceraldehyde-3-phosphate dehydrogenase) mRNA. **(d) **Cells were preincubated in the absence or presence of p38 MAPK inhibitor SB202190 (10 μM) for 30 minutes, followed by incubation by HNE (20 μM) for 48 hours. PGE_2 _secretion was evaluated as described above. Data are means ± standard error of the mean of *n *= 3 and expressed as a percentage of untreated cells. Statistics: Student unpaired *t *test; *p < 0.05, ***p < 0.001. COX-2, cyclooxygenase-2; HNE, 4-hydroxynonenal; MAPK, mitogen-activated protein kinase; PGE_2_, prostaglandin E_2_.

To better characterise the properties of HNE cell signaling in OA osteoblasts, we evaluated *COX-2 *gene expression and PGE_2 _production in response to HNE stimulation. Compared with untreated cells, PGE_2 _level was increased significantly by 209%, 240%, 551%, and 2,434% at concentrations of 1, 5, 10, and 20 μM HNE, respectively (Figure 4a). The increase of PGE_2 _production related directly to an increase in COX-2 protein and mRNA OA osteoblasts. The protein and mRNA levels were increased in a dose-dependent manner by incubation of cells with HNE (0 to 20 μM), with a maximal stimulation at 20 μM HNE (8.5- and 4.6-fold, respectively) (Figure 4b,c).

To delineate the signaling pathways involved in HNE-induced COX-2 expression in pilot experiments, we used cell-permeable chemical inhibitor of p38 MAPK, SB202190. This inhibitor had no effect on the basal PGE_2 _release (data not shown). As shown in Figure 4d, HNE significantly induced PGE_2 _release by 340% in comparison with untreated cells. However, the p38 MAPK inhibitor significantly reduced HNE-stimulated PGE_2 _production. Identical results were obtained with COX-2 protein level (data not shown).

### HNE modulates ALPase, OC, IL-6, and PGE_2 _in normal osteoblasts

**Figure 5:**
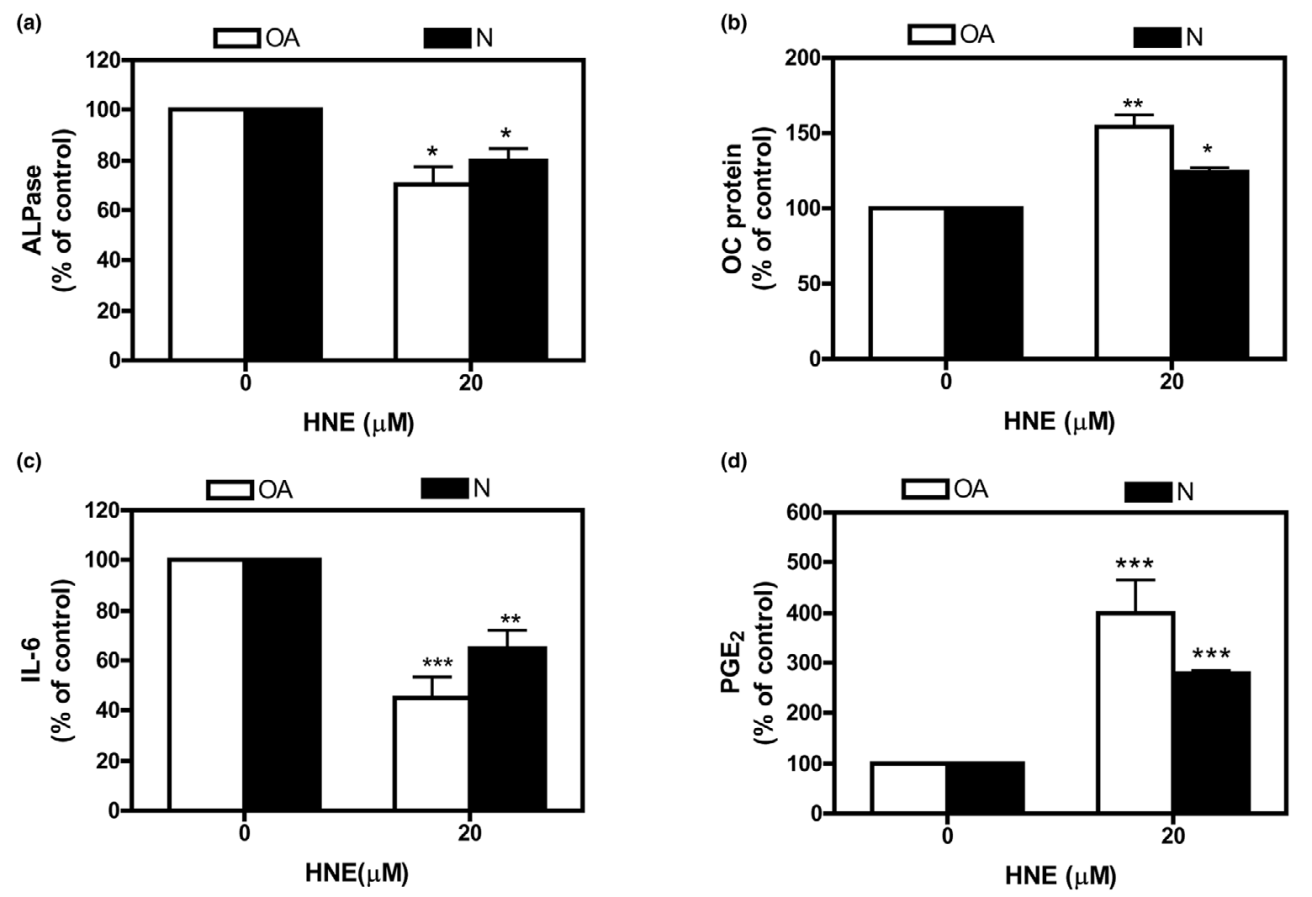
Comparison of the effect of HNE on normal (N) and osteoarthtitic (OA) osteoblast metabolism. Cells were incubated in the absence or presence of 20 μM HNE for 48 hours. ALPase activity **(a) **was determined in cellular extract as described in Materials and methods. OC **(b)**, IL-6 **(c)**, and PGE_2 _**(d) **levels were measured in culture media using specific kits. Data are means ± standard error of the mean of *n *= 3 and expressed as a percentage of untreated cells. Statistics: Student unpaired *t *test; *p < 0.05, **p < 0.01, ***p < 0.001. ALPase, alkaline phosphatase; HNE, 4-hydroxynonenal; IL-6, interleukin-6; OC, osteocalcin; PGE_2_, prostaglandin E_2_.

Next, we examined whether HNE can also modulate the activity of ALPase as well as the production of OC, PGE_2_, and IL-6 in normal osteoblasts. In a similar pattern, our data showed that HNE at 20 μM inhibits ALPase activity (Figure 5a) and IL-6 production (Figure 5c) but, in contrast, induced OC (Figure 5b) and PGE_2 _(Figure 5d) release.

### HNE activates p38 MAPK and JNK1/2, but not ERK1/2

**Figure 6:**
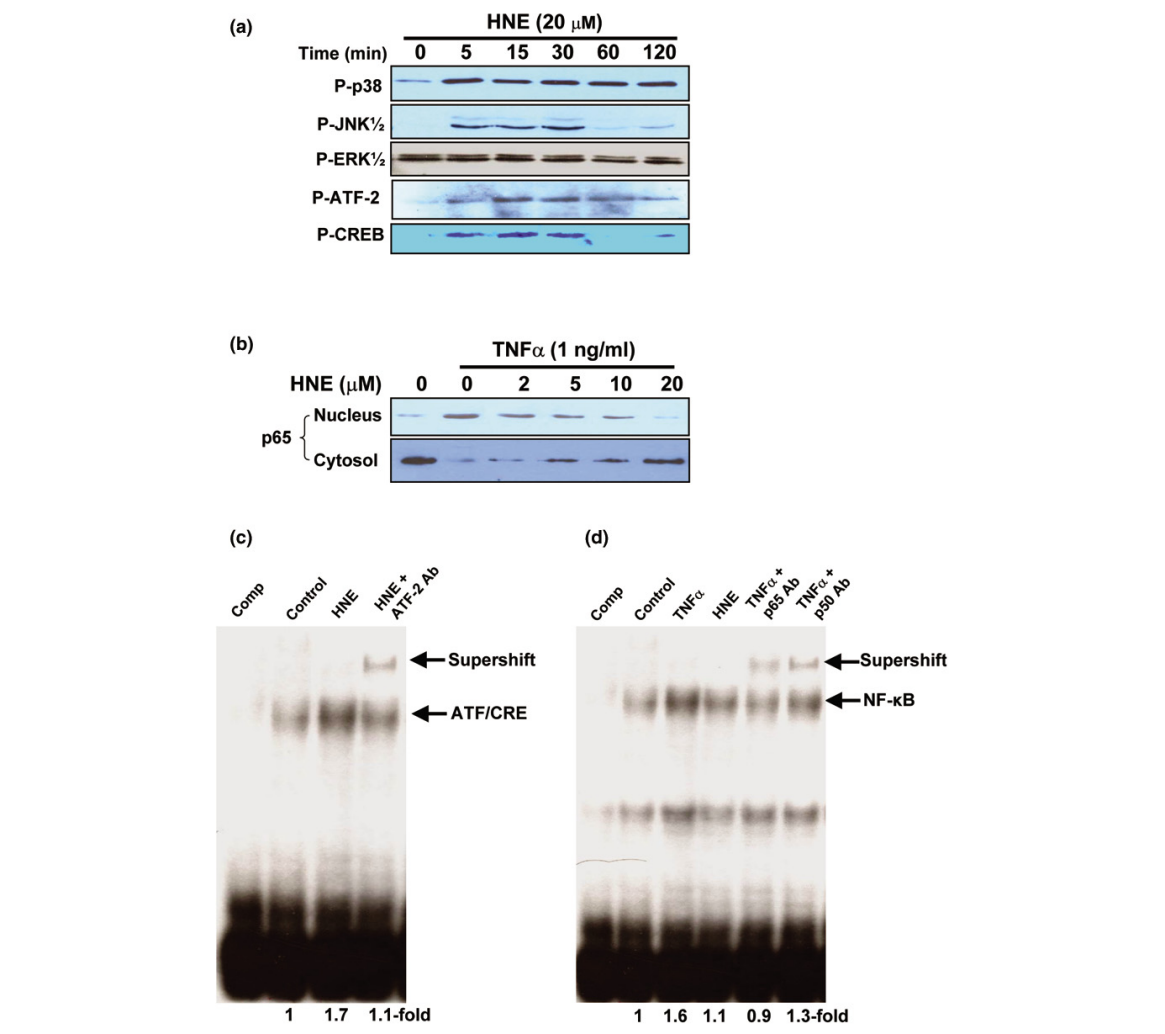
Effect of HNE on signaling pathways. **(a, b) **Osteoblasts were treated with 20 μM HNE for the indicated times in the presence or absence of 1 ng/ml TNF-α. Total cell lysates or nuclear extracts (approximately 50 μg) were prepared and subjected to Western analysis with anti-phosphospecific antibodies anti-phospho-p38 MAPK, anti-phospho-JNK1/2, anti-phospho-ERK1/2, anti-phospho-ATF-2 and anti-phospho-CREB-1, and anti-NF-κB/p65. **(c, d) **Osteoblasts were incubated in absence (control) or presence of 1 ng/ml TNF-α, 20 μM HNE, or 20 μM HNE combined with 1 ng/ml TNF-α in serum-free medium for 1 hour. Nuclear extracts were prepared and subjected to electrophoretic mobility shift assay using ATF/CRE **(c) **and NF-κB **(d) **oligonucleotide probes. Specificity of the binding was assayed by competition (comp) of the oligonucleotide with 50-fold of excess unlabeled ATF/CRE or NF-κB oligonucleotide or by the adding specific antibodies anti-ATF-2, anti-p50, or anti-p65. Arrows refer to specific DNA–protein complex. Data are representative of three to five independent expriments. ATF-2, activating transcription factor-2; CREB-1, CRE-binding factor-1; ERK, extracellular signal-regulated kinase; HNE, 4-hydroxynonenal; JNK, c-Jun NH_2_-terminal kinase; MAPK, mitogen-activated protein kinase; NF-κB, nuclear factor-κB; TNF-α, tumour necrosis factor-α.

To gain insight into the signaling pathway activated by HNE in human OA osteoblasts, we first examined the HNE-induced phosphorylation patterns of MAPKs over increasing periods of time. Our data indicated that HNE stimulated p38 MAPK phosphorylation within 5 minutes and remained in a phosphorylated state for 120 minutes (Figure 6a). JNK2 (p46) was phosphorylated in a time-dependent manner, reaching a maximum between 5 and 30 minutes and returning to the basal level at 60 minutes. HNE had no effect on the ERK1/2 phosphorylation levels. No change in the total protein level of MAPKs was noted (data not shown).

### HNE induced ATF-2/CREB activation but inhibited NF-κB

Next, we investigated the effect of HNE on p38 MAPK downstream transcription factors CREB-1 and ATF-2. Our data showed that exposure of 20 μM HNE resulted in an early phosphorylation of ATF-2 and CREB-1 after 5 minutes of incubation (Figure 6a). We also examined the effect of HNE on the total NF-κB/p65 and phosphorylated IκBα. Our data showed that HNE had no significant effect on the basal level of the phosphorylated IκBα and cytosolic and nuclear NF-κB/p65 in osteoblasts (data not shown). However, combined with TNF-α, HNE inhibited strongly NF-κB/p65 protein translocation in the nucleus in a dose-dependent manner (Figure 6b).

### HNE increased DNA binding of ATF/CRE, but decreased DNA binding of NF-κB

To explore the effect of HNE on DNA-binding activity of ATF/CRE and NF-κB, OA osteoblasts were incubated for 60 minutes with 20 μM HNE or 1 ng/ml TNF-α. The latter was used as a positive control of NF-κB activation. EMSA data showed that HNE increased the DNA-binding activity of ATF/CRE to 170% compared with unstimulated cells (Figure 6c). However, TNF-α (but not HNE) induced the DNA-binding activity of NF-κB by 160% (Figure 6d). Basal and induced binding was displaced by adding 50-fold excess cold ATF/CRE and NF-κB oligonucleotide (competition).

To further identify the ATF/CRE and NF-κB protein complexes that bind on these motifs, specific anti-ATF-2, anti-p65, and anti-p55 antibodies were added to the shift reaction mixture. As illustrated in Figure 6c,d, the ATF-2 was supershifted in HNE-treated osteoblasts, and the p65 and p50 proteins were supershifted in TNF-α-treated osteoblasts.

### HNE induced COX-2 promoter activity via CRE site

**Figure 7:**
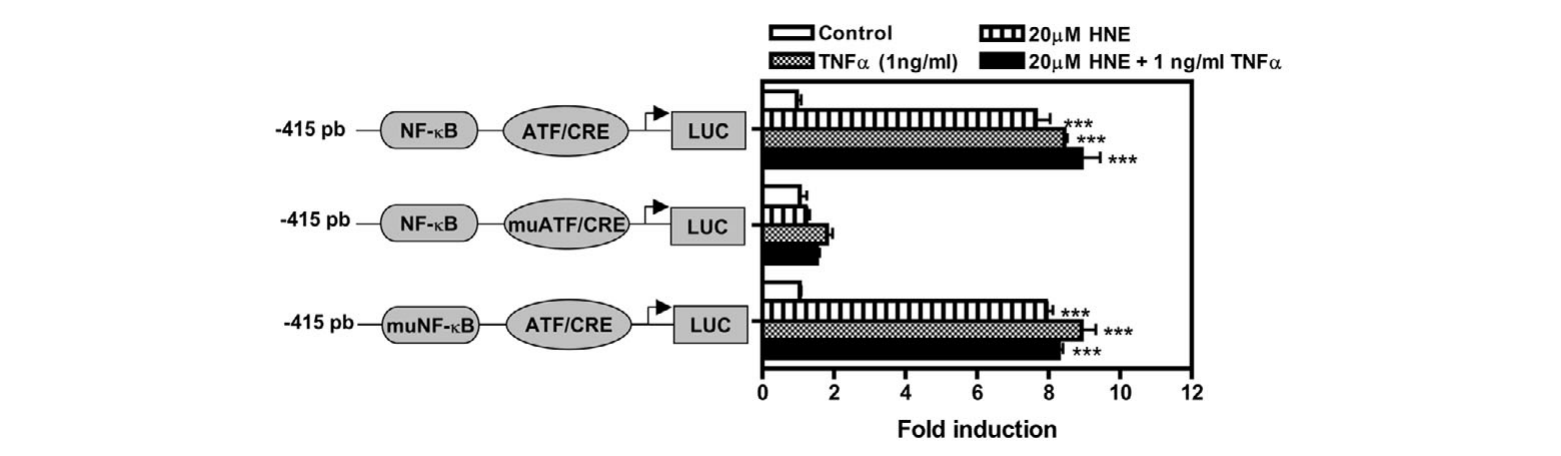
Functional analysis of COX-2 promoter in MG-63 osteoblast-like line cells. The -415 constructs of the COX-2 promoter fused to a Luciferase (Luc) reporter gene, its mutated ATF/CRE derivative (muATF/CRE), and mutated NF-κB derivative (muNF-κB) are shown in schematic representation. The constructs were co-transfected in MG-63 osteoblast-like line cells with pCMV-β-galactosidase (pCMV-β-gal). Six hours after transfection, fresh 0.5% foetal bovine serum/Dulbecco's modified Eagle's medium was added in the absence or presence of 20 μM HNE, 1 ng/ml TNF-α, and 20 μM HNE + 1 ng/ml TNF-α for another 24 hours. The β-gal and Luc levels were then measured in cellular extracts using specific commercial kits, and data were normalised for Luc and β-gal activities. Values are mean ± standard error of the mean of three experiments. Statistics: *p *values determined by Student unpaired *t *test: ***p < 0.001. *P *values are versus autologous untreated cells (control). COX-2, cyclooxygenase-2; HNE, 4-hydroxynonenal; NF-κB, nuclear factor-κB; TNF-α, tumour necrosis factor-α.

To examine for elements of transcriptional control of the *COX-2 *gene stimulated by HNE, we conducted transient transfection analyses with a WT (-415)-Luc COX-2 promoter construct harbouring enhancer elements [21-23] for critical transcription factors, including an ATF/CRE site (-58 to -53). Data showed that HNE (20 μM) as well as TNF-α (1 ng/ml) upregulated the COX-2 promoter activity by 7.8- and 8.4-fold, respectively, compared with control (Figure 7). The mutation of the ATF/CRE site decreased the basal COX-2 promoter activity. In addition, their inducibility by either HNE or TNF-α was completely abrogated. However, the mutation of proximal NF-κB site (-223/-214) in the human COX-2 promoter construct was without effect in terms of basal and HNE-stimulated luciferase activity.

### IL-6 and PGE_2 _modulation by HNE is related to IKKα and p38 MAPK signaling pathways, respectively

**Figure 8:**
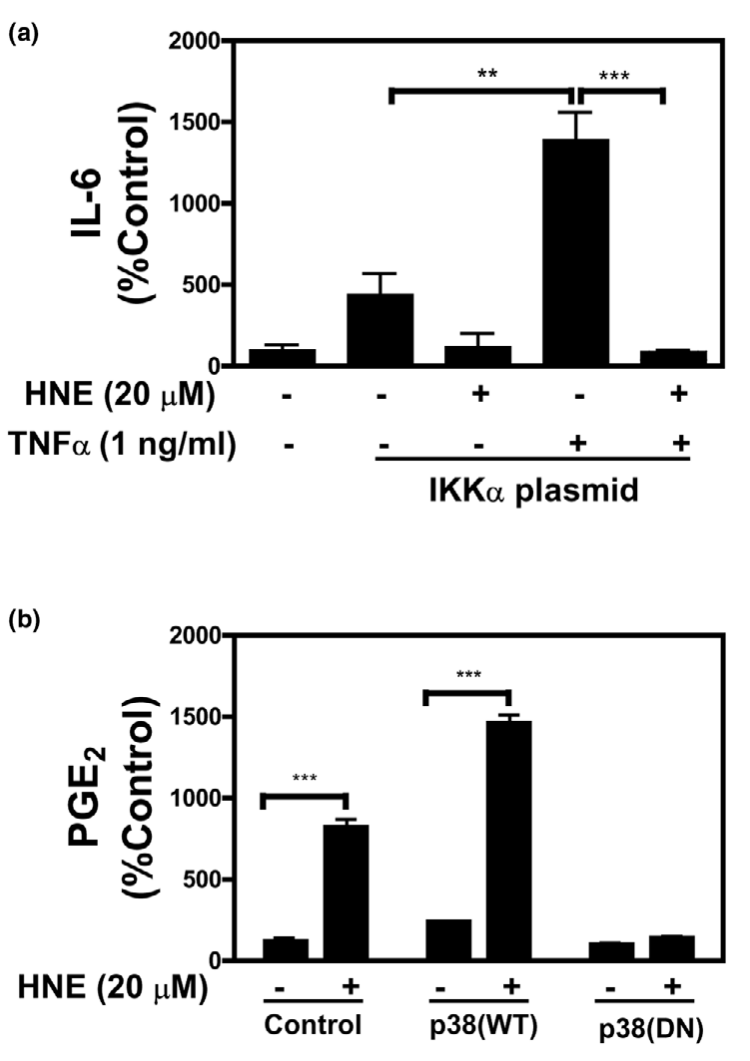
Effect of IKKα and p38 MAPK overexpression on HNE-regulated IL-6 and PGE_2 _release in MG-63 osteoblast-like line cells. Transient transfection of MG-63 osteoblasts was performed with 1 μg of expression vectors of IKKα **(a)**, WT, or DN p38 MAPK **(b) **as described in Materials and methods. Six hours after transfection, fresh 0.5% foetal bovine serum/Dulbecco's modified Eagle's medium was added in the absence or presence of 20 μM HNE, 1 ng/ml TNF-α, and 20 μM HNE + 1 ng/ml TNF-α for another 24 hours. After incubation, culture medium was collected and IL-6 and PGE_2 _levels were evaluated by commercial kits. Values are mean ± standard error of the mean of three experiments. Statistics: *p *values determined by Student unpaired *t *test. *P *values are versus autologous untreated cells or TNF-α-treated cells as indicated in the figure. **p < 0.01, ***p < 0.001. DN, dominant negative; HNE, 4-hydroxynonenal; IKKα, IkappaB kinase alpha; IL-6, interleukin-6; MAPK, mitogen-activated protein kinase; PGE_2_, prostaglandin E_2_; TNF-α, tumour necrosis factor-α; WT, wild-type.

Finally, for a better understanding of the role of IKKα in NF-κB-mediated IL-6 production, constitutive activated IKKα was overexpressed in MG-63 osteoblast-like cells and then cells were incubated with TNF-α, HNE, or TNF-α combined with HNE. Our data showed that IKKα overexpression stimulated IL-6 production in the presence of 1 ng/ml TNF-α, an effect completely abrogated by HNE (Figure 8a). These results indicated that the inhibition of the IKKα pathway is the major regulator of the IL-6 response to HNE.

To further confirm that p38 MAPK plays a principal role in mediating HNE-induced COX-2 in osteoblasts, we transfected expression vectors of WT p38 MAPK and DN p38 MAPK followed by HNE stimulation. Overexpression of WT p38 plasmid markedly increased PGE_2 _production, and HNE treatment further enhanced PGE_2 _release compared with control cells (Figure 8b). However, the overexpression of DN p38 MAPK abrogated this effect.

## Discussion

This study was aimed at clarifying the regulation of OA osteoblast activity by HNE, a very reactive aldehyde produced during ROS-induced LPO. One major finding of this study was an alteration in the production of ALPase, OC, and Col I by osteoblasts after HNE exposure. Also, HNE up to 20 μM did not alter the cell viability but at 50 μM was cytotoxic and significantly decreased the cell viability (approximately 40%) compared with untreated cells (data not shown). Based on these data, all subsequent experiments were conducted using HNE up to 20 μM. The mechanism of HNE cytotoxicity was demonstrated in various cell types and tissues and is believed to be related to the chemical modification of cellular proteins by HNE. Among a number of proteins modified by HNE, citric acid cycle enzymes and cytochrome C oxidase were detected as the major targets of HNE in cells[5,24,25]. On the other hand, exposure of cells to HNE resulted in rapid reduction of cellular glutathione levels, suggesting that HNE influences primarily the redox status of the cells[26].

Firstly, we demonstrated that HNE (at ≤10 μM) reduced ALPase activity without changing its expression. These data suggest the existence of a post-translational mechanism that decreases ALPase activity, possibly through HNE binding. This is based on previous reports showing that H_2_O_2 _and glucose mediate post-translational modification of this enzyme and, in turn, enzyme inactivation[27,28]. Because bone ALPase is a transmembrane protein, and the membrane is a source of HNE production by LPO process, we suggest that ALPase would be more susceptible to attack by this aldehyde. With the ultimate goal of determining whether ALPase was a target for HNE, we have incubated bovine recombinant ALPase with increasing concentrations of HNE. Our preliminary data showed that this aldehyde inhibits enzyme activity in a dose-dependent manner (study in progress). Given that ALPase is one of the markers of osteoblast phenotypic differentiation and plays an important role in bone formation, the observed decrease in its activity in response to HNE exposure supports the hypothesis that this molecule prevents osteoblast differentiation/mineralisation. Our data are in agreement with those of Parhami and colleagues [29] and Mody and colleagues [30], showing that lipid oxidation products inhibit osteoblastic differentiation of marrow stromal cells as demonstrated by inhibition of ALPase activity. ALPase participates in the differentiation of osteoblasts and provides phosphate for hydroxyapatite mineral formation. Its inhibition by HNE could therefore lead to impaired bone mineralisation[31].

Secondly, in contrast to ALPase, OC expression at protein and mRNA levels was significantly increased after treatment with HNE (1 to 20 μM). OC is synthesised predominately by osteoblasts and represents the most osteoblast-specific gene. Glowacki and colleagues [10] supported the hypothesis that OC may function as a matrix signal in the recruitment and differentiation of bone-resorbing cells. Because OC is limited to the osteoblast, analysis of its expression *in vitro *provides important information about terminal osteoblast differentiation [32]. Increased bone formation is observed in OC knockout mice[33]. These findings underline the importance of OC in bone turnover, suggesting that OC retards bone formation/mineralisation[34]. Therefore, the increased OC levels in HNE-treated osteoblasts indicate that HNE plays an important role in regulation of osteoblastic bone formation functions.

Thirdly, we demonstrated that HNE induces Col I α1 expression in human osteoblasts at the protein, but not at the mRNA, level. This may indicate that HNE upregulates Col I synthesis at the post-transcriptional step, but we cannot explain this finding at this time. Further investigations will be performed to explain why HNE does not affect the mRNA level of Col I by determining RNA-binding proteins. Among them, alphaCP protein was identified as having a critical role in mRNA stabilisation of Col I. The induction of Col I synthesis by HNE in osteoblasts suggests that this aldehyde contributes to the elevation of collagen deposition in OA bone and supports other reports. Parola and colleagues [35] were the first to demonstrate that HNE upregulates Col I in human hepatic fat-storing cells. In another study, Garcia-Ruiz and colleagues [36] demonstrated that MDA upregulates Col I through activation of Sp1 transcription factor. It has been reported that HNE acted as a potent pro-fibrogenic stimuli in the expression of genes involved in ECM deposition in hepatic fat-storing cells [35]. Nevertheless, our findings that HNE potentially acts as a pro-fibrogenic stimulus to induce Col I production in osteoblasts suggest a possible link between oxidant stress and OA trabecular subchondral bone fibrosis.

In our study, we considered it essential to address several potential factors, such as IL-6 and PGE_2_, that play a critical role in bone resorption. Our previous study reported that OA subchondral and trabecular osteoblasts produce more IL-6 and PGE_2 _levels than normal cells[37]. We demonstrate here that HNE inhibits basal and TNF-α-induced IL-6 expression at both protein and mRNA levels in osteoblasts via the NF-κB signaling pathway. We have detected significant changes in total IκBα and a slight decrease of phosphorylated IκBα. Moreover, no translocation of NF-κB/p65 from cytosol to the nucleus was observed in HNE-treated osteoblasts. This observation was confirmed by NF-κB-binding activity and IKKα overexpression. We demonstrated that HNE inhibits TNF-α-induced NF-κB binding. Interestingly, HNE completely blocked the IKKα-enhanced TNF-α-induced IL-6 production. These data are consistent with other studies suggesting that HNE exerts its inhibitory action at the IKKα level or upstream, thereby affecting subsequent IκBα phosphorylation/proteolysis[38]. The inhibition of NF-κB system by HNE and prevention of degradation of IκBα are associated with certain alterations of expression of the NF-κB target gene product, such as inducible nitric oxide synthase [39] and IL-6[40]. As indicated, the regulation of IL-6 expression is governed predominantly by the ubiquitously expressed transcription factor NF-κB, which is required for the inducible expression of genes associated with inflammatory responses[41]. The report of Dendorfer and colleagues [42] indicated that mutations in the NF-κB site of IL-6 promoter completely abolished lipopolysaccharide-induced promoter activity in murine monocyte-macrophage cell line PU5-1.8. Altogether, our findings combined with the existing literature indicate that HNE inhibits IL-6 expression at the transcriptional level via NF-κB signaling pathways.

COX-2 and PGE_2 _levels were markedly increased in osteoblasts treated with HNE. We demonstrated that the p38 MAPK pathway played a key role in the mechanism of HNE-induced PGE_2 _expression. We observed that HNE stimuli elicited a rapid and significant phosphorylation of p38 MAPK in osteoblasts. Next, we tested the effect of a p38 MAPK-specific inhibitor, SB202190, on PGE_2 _release. Our data showed that SB202190 blocked completely the HNE-induced PGE_2 _production. Furthermore, the overexpression of WT p38 MAPK enhanced PGE_2 _expression, and conversely DN p38 MAPK decreased HNE-induced PGE_2 _levels. Kumagai and colleagues[43] have proposed for the first time the implication of p38 MAPK in HNE-induced COX-2 in epithelial cells. The authors have demonstrated that HNE enhances COX-2 expression by the stabilisation of COX-2 mRNA via the p38 MAPK pathway. In COX-2 promoter, numerous cis-elements are identified to exert transcriptional control of COX-2[22,44]. Among these elements, ATF/CRE was shown to act as the most critical of these regulatory elements for COX-2 transcription[21]. Mutation in the ATF/CRE sequence attenuates HNE-stimulated COX-2 promoter activity. Mutating the more proximal NF-κB in the human COX-2 promoter construct was without effect in the basal and HNE-stimulated luciferase activity, suggesting that the NF-κB site in the promoter region of *COX-2 *gene is not involved in the HNE-induced COX-2 expression. In many cell types, the ATF/CRE site is activated by homodimers and heterodimers of c-Jun, c-Fos, and ATF/CRE family members subsequent to serum, 12-*O*-tetradecanoylphorbol-13-acetate, or growth factor stimulation. HNE was shown to induce ATF-2 and CREB-1 phosphorylation and DNA-binding activity of the ATF/CRE site. Under different cytokines and growth factors, NF-κB and ERK1/2 are known regulators of COX-2 expression[45,46]. However, neither IκBα degradation nor translocalisation of NF-κB from cytoplasm to the nucleus was observed by western blotting analysis and ERK1/2 kinase was not phosphorylated by HNE treatment. This observation indicates that NF-κB and ERK1/2 are not involved in the HNE-induced COX-2 expression.

## Conclusion

In this study, we identified for the first time a novel mechanism linking oxidative stress to nuclear signaling in OA osteoblasts through the action of HNE, an LPO end product. Our data suggest that HNE may contribute in OA development via its ability to alter cellular phenotype and metabolic activity of osteoblasts. In the light of the previous data on increased HNE levels in OA articular tissues, particular interest should be addressed to the pathophysiological role of this aldehyde in OA.

## Abbreviations

ALPase = alkaline phosphatase; ATF-2 = activating transcription factor-2; Col I = type I collagen; COX-2 = cyclooxygenase-2; CREB-1 = CRE-binding factor-1; C_T _= threshold cycle; DN = dominant negative; ECM = extracellular matrix; ELISA = enzyme-linked immunosorbent assay; ERK = extracellular signal-regulated kinase; FBS = foetal bovine serum; GAPDH = glyceraldehyde-3-phosphate dehydrogenase; HNE = hydroxynonenal; IKKα = IkappaB kinase alpha; IL-6 = interleukin-6; JNK = c-Jun NH_2_-terminal kinase; MAPK = mitogen-activated protein kinase; MDA = malondialdehyde; NF-κB = nuclear factor-κB; OA = osteoarthritic; OC = osteocalcin; LPO = lipid peroxidation; PCR = polymerase chain reaction; PGE_2 _= prostaglandin E_2_; ROS = reactive oxygen species; TNF-α = tumour necrosis factor-α; TTBS = 20 mM Tris, pH7.4, 150 mM NaCl, 0.1% Tween 20; UNG = uracil-N-glycosylase; WT = wild-type.

## Competing interests

The authors declare that they have no competing interests.

## Authors' contributions

QS carried out the experimental study, contributed to the preparation of the manuscript, and performed statistical analysis. FV and VC assisted in the experiments and in the isolation of osteoblasts from human bone. PL and HF evaluated and interpreted data and assisted with the preparation of the manuscript. HA assisted in the real-time PCR experiments. JADB cloned the COX-2 promoter constructs and contributed to manuscript preparation. JCF assisted with the design of experiments and obtained human tissues. MB designed the study, supervised the project, evaluated and interpreted data, and prepared the manuscript. All authors read and approved the final manuscript.

## References

[B1] Uchida K (2003). 4-Hydroxy-2-nonenal: a product and mediator of oxidative stress. Prog Lipid Res.

[B2] Esterbauer H, Schaur RJ, Zollner H (1991). Chemistry and biochemistry of 4-hydroxynonenal, malonaldehyde and related aldehydes. Free Radic Biol Med.

[B3] Page S, Fischer C, Baumgartner B, Haas M, Kreusel U, Loidl G, Hayn M, Ziegler-Heitbrock HW, Neumeier D, Brand K (1999). 4-Hydroxynonenal prevents NF-kappaB activation and tumor necrosis factor expression by inhibiting IkappaB phosphorylation and subsequent proteolysis. J Biol Chem.

[B4] Uchida K, Shiraishi M, Naito Y, Torii Y, Nakamura Y, Osawa T (1999). Activation of stress signaling pathways by the end product of lipid peroxidation. 4-hydroxy-2-nonenal is a potential inducer of intracellular peroxide production. J Biol Chem.

[B5] Benderdour M, Charron G, Deblois D, Comte B, Des Rosiers C (2003). Cardiac mitochondrial NADP^+^-isocitrate dehydrogenase is inactivated through 4-hydroxynonenal adduct formation: an event that precedes hypertrophy development. J Biol Chem.

[B6] Benderdour M, Charron G, Comte B, Ayoub R, Beaudry D, Foisy S, Deblois D, Des Rosiers C (2004). Decreased cardiac mitochondrial NADP^+^-isocitrate dehydrogenase activity and expression: a marker of oxidative stress in hypertrophy development. Am J Physiol Heart Circ Physiol.

[B7] Grigolo B, Roseti L, Fiorini M, Facchini A (2003). Enhanced lipid peroxidation in synoviocytes from patients with osteoarthritis. J Rheumatol.

[B8] Morquette B, Shi Q, Lavigne P, Ranger P, Fernandes JC, Benderdour M (2002). Production of lipid peroxidation products in osteoarthritic tissues: new evidence linking 4-hydroxynonenal to cartilage degradation. Arthritis Rheum.

[B9] Lajeunesse D, Reboul P (2003). Subchondral bone in osteoarthritis: a biologic link with articular cartilage leading to abnormal remodeling. Curr Opin Rheumatol.

[B10] Glowacki J, Rey C, Glimcher MJ, Cox KA, Lian J (1991). A role for osteocalcin in osteoclast differentiation. J Cell Biochem.

[B11] Shi Q, Lajeunesse D, Reboul P, Martel-Pelletier J, Pelletier JP, Dehnade F, Fernandes JC (2002). Metabolic activity of osteoblasts from periprosthetic trabecular bone in failed total hip arthroplasties and osteoarthritis as markers of osteolysis and loosening. J Rheumatol.

[B12] Dubois RN, Abramson SB, Crofford L, Gupta RA, Simon LS, Van De Putte LB, Lipsky PE (1998). Cyclooxygenase in biology and disease. FASEB J.

[B13] Miller SC, Marks SC (1994). Effects of prostaglandins on the skeleton. Clin Plast Surg.

[B14] Smith PK, Krohn RI, Hermanson GT, Mallia AK, Gartner FH, Provenzano MD, Fujimoto EK, Goeke NM, Olson BJ, Klenk DC (1985). Measurement of protein using bicinchoninic acid. Anal Biochem.

[B15] Trentz OA, Hoerstrup SP, Sun LK, Bestmann L, Platz A, Trentz OL (2003). Osteoblasts response to allogenic and xenogenic solvent dehydrated cancellous bone *in vitro*. Biomaterials.

[B16] Bernard MP, Chu ML, Myers JC, Ramirez F, Eikenberry EF, Prockop DJ (1983). Nucleotide sequences of complementary deoxyribonucleic acids for the pro alpha 1 chain of human type I procollagen. Statistical evaluation of structures that are conserved during evolution. Biochemistry.

[B17] Ishimi Y, Miyaura C, Jin CH, Akatsu T, Abe E, Nakamura Y, Yamaguchi A, Yoshiki S, Matsuda T, Hirano T (1990). IL-6 is produced by osteoblasts and induces bone resorption. J Immunol.

[B18] Origuchi T, Migita K, Nakashima T, Honda S, Yamasaki S, Hida A, Kawakami A, Aoyagi T, Kawabe Y, Eguchi K (2000). Regulation of cyclooxygenase-2 expression in human osteoblastic cells by N-acetylcysteine. J Lab Clin Med.

[B19] Reinecke P, Knopf C, Schmitz M, Schneider EM, Gabbert HE, Gerharz CD (2000). Growth inhibitory effects of paclitaxel on human epithelioid sarcoma *in vitro*: heterogeneity of response and the multidrug resistance phenotype. Cancer.

[B20] Benderdour M, Tardif G, Pelletier JP, Di Battista JA, Reboul P, Ranger P, Martel-Pelletier J (2002). Interleukin 17 (IL-17) induces collagenase-3 production in human osteoarthritic chondrocytes via AP-1 dependent activation: differential activation of AP-1 members by IL-17 and IL-1beta. J Rheumatol.

[B21] Faour WH, Mancini A, He QW, Di Battista JA (2003). T-cell-derived interleukin-17 regulates the level and stability of cyclooxygenase-2 (COX-2) mRNA through restricted activation of the p38 mitogen-activated protein kinase cascade: role of distal sequences in the 3'-untranslated region of COX-2 mRNA. J Biol Chem.

[B22] Appleby SB, Ristimaki A, Neilson K, Narko K, Hla T (1994). Structure of the human cyclo-oxygenase-2 gene. Biochem J.

[B23] Smith WL, Dewitt DL, Garavito RM (2000). Cyclooxygenases: structural, cellular, and molecular biology. Annu Rev Biochem.

[B24] Musatov A, Carroll CA, Liu YC, Henderson GI, Weintraub ST, Robinson NC (2002). Identification of bovine heart cytochrome c oxidase subunits modified by the lipid peroxidation product 4-hydroxy-2-nonenal. Biochemistry.

[B25] Humphries KM, Szweda LI (1998). Selective inactivation of alpha-ketoglutarate dehydrogenase and pyruvate dehydrogenase: reaction of lipoic acid with 4-hydroxy-2-nonenal. Biochemistry.

[B26] Liu W, Kato M, Akhand AA, Hayakawa A, Suzuki H, Miyata T, Kurokawa K, Hotta Y, Ishikawa N, Nakashima I (2000). 4-hydroxynonenal induces a cellular redox status-related activation of the caspase cascade for apoptotic cell death. J Cell Sci.

[B27] Koyama I, Yakushijin M, Goseki M, Iimura T, Sato T, Sonoda M, Hokari S, Komoda T (1998). Partial breakdown of glycated alkaline phosphatases mediated by reactive oxygen species. Clin Chim Acta.

[B28] Sok DE (1999). Oxidative inactivation of brain alkaline phosphatase responsible for hydrolysis of phosphocholine. J Neurochem.

[B29] Parhami F, Basseri B, Hwang J, Tintut Y, Demer LL (2002). High-density lipoprotein regulates calcification of vascular cells. Circ Res.

[B30] Mody N, Parhami F, Sarafian TA, Demer LL (2001). Oxidative stress modulates osteoblastic differentiation of vascular and bone cells. Free Radic Biol Med.

[B31] Kartsogiannis V, Ng KW (2004). Cell lines and primary cell cultures in the study of bone cell biology. Mol Cell Endocrinol.

[B32] Handschin AE, Trentz OA, Hoerstrup SP, Kock HJ, Wanner GA, Trentz O (2005). Effect of low molecular weight heparin (dalteparin) and fondaparinux (Arixtra) on human osteoblasts *in vitro*. Br J Surg.

[B33] Wolf G (1996). Function of the bone protein osteocalcin: definitive evidence. Nutr Rev.

[B34] Ducy P, Zhang R, Geoffroy V, Ridall AL, Karsenty G (1997). Osf2/Cbfa1: a transcriptional activator of osteoblast differentiation. Cell.

[B35] Parola M, Pinzani M, Casini A, Albano E, Poli G, Gentilini A, Gentilini P, Dianzani MU (1993). Stimulation of lipid peroxidation or 4-hydroxynonenal treatment increases procollagen alpha 1 (I) gene expression in human liver fat-storing cells. Biochem Biophys Res Commun.

[B36] Garcia-Ruiz I, de la Torre P, Diaz T, Esteban E, Fernandez I, Munoz-Yague T, Solis-Herruzo JA (2002). Sp1 and Sp3 transcription factors mediate malondialdehyde-induced collagen alpha 1(I) gene expression in cultured hepatic stellate cells. J Biol Chem.

[B37] Massicotte F, Lajeunesse D, Benderdour M, Pelletier JP, Hilal G, Duval N, Martel-Pelletier J (2002). Can altered production of interleukin-1beta, interleukin-6, transforming growth factor-beta and prostaglandin E(2) by isolated human subchondral osteoblasts identify two subgroups of osteoarthritic patients. Osteoarthritis Cartilage.

[B38] Valacchi G, Pagnin E, Phung A, Nardini M, Schock BC, Cross CE, van der Vliet A (2005). Inhibition of NFkappaB activation and IL-8 expression in human bronchial epithelial cells by acrolein. Antioxid Redox Signal.

[B39] Liu W, Kato M, Itoigawa M, Murakami H, Yajima M, Wu J, Ishikawa N, Nakashima I (2001). Distinct involvement of NF-kappaB and p38 mitogen-activated protein kinase pathways in serum deprivation-mediated stimulation of inducible nitric oxide synthase and its inhibition by 4-hydroxynonenal. J Cell Biochem.

[B40] Luckey SW, Taylor M, Sampey BP, Scheinman RI, Petersen DR (2002). 4-hydroxynonenal decreases interleukin-6 expression and protein production in primary rat Kupffer cells by inhibiting nuclear factor-kappaB activation. J Pharmacol Exp Ther.

[B41] Vanden BW, Vermeulen L, De WG, De BK, Boone E, Haegeman G (2000). Signal transduction by tumor necrosis factor and gene regulation of the inflammatory cytokine interleukin-6. Biochem Pharmacol.

[B42] Dendorfer U, Oettgen P, Libermann TA (1994). Multiple regulatory elements in the interleukin-6 gene mediate induction by prostaglandins, cyclic AMP, and lipopolysaccharide. Mol Cell Biol.

[B43] Kumagai T, Nakamura Y, Osawa T, Uchida K (2002). Role of p38 mitogen-activated protein kinase in the 4-hydroxy-2-nonenal-induced cyclooxygenase-2 expression. Arch Biochem Biophys.

[B44] Yamamoto K, Arakawa T, Ueda N, Yamamoto S (1995). Transcriptional roles of nuclear factor kappa B and nuclear factor-interleukin-6 in the tumor necrosis factor alpha-dependent induction of cyclooxygenase-2 in MC3T3-E1 cells. J Biol Chem.

[B45] Wu D, Marko M, Claycombe K, Paulson KE, Meydani SN (2003). Ceramide-induced and age-associated increase in macrophage COX-2 expression is mediated through up-regulation of NF-kappa B activity. J Biol Chem.

[B46] Jones MK, Sasaki E, Halter F, Pai R, Nakamura T, Arakawa T, Kuroki T, Tarnawski AS (1999). HGF triggers activation of the COX-2 gene in rat gastric epithelial cells: action mediated through the ERK2 signaling pathway. FASEB J.

